# Pathways, Mechanisms, and Therapeutic Strategies of Neurotoxicity Induced by Micro- and Nanoplastics

**DOI:** 10.3390/brainsci15121345

**Published:** 2025-12-18

**Authors:** Min Yan, Yanfeng Chen, Ying Tao, Hui Wang, Xuewen Tian, Xiuxiu Wang

**Affiliations:** 1 Academy of Sports Sciences, Shandong Sport University, Jinan 250102, China; 202520200585@stu.tjus.edu.cn (M.Y.); chenyanfeng@stu.sdpei.edu.cn (Y.C.); taoying@stu.sdpei.edu.cn (Y.T.); wanghui@stu.sdpei.edu.cn (H.W.); tianxuewen@sdpei.edu.cn (X.T.); 2 School of Sports and Health, Tianjin Sport University, Tianjin 301617, China

**Keywords:** neurotoxicity, microplastics, nanoplastics, environmental pollution, therapeutic interventions, central nervous system

## Abstract

Plastic pollution now represents a global environmental crisis, as micro- and nanoplastics (MNPs) infiltrate organisms via multiple routes such as the digestive system and respiratory system, ultimately accumulating in tissues and endangering human health. The central nervous system exhibits particular vulnerability to MNPs toxicity, which can trigger neurotoxic effects and disrupt brain function, potentially contributing to neurological disorders. Understanding the precise mechanisms and biological pathways underlying MNP-induced neurotoxicity has therefore emerged as a critical step toward identifying therapeutic targets. This review synthesizes current knowledge on MNPs’ entry routes into the brain, examines proposed neurotoxic mechanisms, and evaluates existing and prospective treatment approaches. These insights may inform both the risk assessment of MNPs exposure and the development of targeted interventions for neurological protection.

## 1. Introduction

Plastic pollution now represents a pressing global environmental crisis [1]. Through processes like photodegradation, oxidation, hydrolysis, and mechanical fragmentation, plastics break down into microplastics (MPs) and nanoplastics (NPs). Their environmental persistence, stemming from non-biodegradability and widespread dispersal, has raised substantial concern [2]. These particles contaminate aquatic, terrestrial, and atmospheric systems while also infiltrating organisms via digestive system and respiration, ultimately bioaccumulating in human tissues [3,4]—a phenomenon threatening both ecosystem integrity and public health. Direct epidemiological evidence regarding the health impacts of MNPs on humans remains extremely limited. However, recent evidence indicates sustained human exposure to foodborne MNPs, supported by existing data indicating that “over 690 marine species have been contaminated by MNPs” [5]. This is because these substances tend to accumulate within the food chain and transfer to organisms at higher trophic levels, including humans [6].

Micro- and Nanoplastics (MNPs) gain systemic access through ingestion, inhalation, and dermal absorption [7,8], distributing across multiple organs and triggering toxic, inflammatory, and oxidative responses in neural, pulmonary, hepatic, and renal tissues [9]. Clinical studies have detected MNPs in human placental, pulmonary and blood samples, with particle sizes ranging from 0.3 to 3000 μm [10,11,12]. The central nervous system (CNS) appears particularly susceptible to MNP toxicity [13]. Experimental evidence demonstrates dose-dependent MNP accumulation in murine brains alongside increased blood–brain barrier (BBB) permeability [9]. Such findings implicate MNPs in neurological dysfunction, with proposed mechanisms including BBB disruption, immunocyte activation, oxidative damage, and membrane interference [14,15,16]. Emerging research further reveals novel pathways like nasopharyngeal transport and neurotransmitter modulation [17,18], underscoring gaps in understanding MNPs neurotoxicology. Therapeutic interventions for MNP-induced neural damage remain exploratory, with pharmacological targets largely uncharacterized.

Collectively, MNPs pose multisystem health risks, with disproportionate neurological consequences potentially linked to neurodegeneration. Elucidating their neurotoxic mechanisms proves critical for risk assessment, preventive policy formulation, and therapeutic development [19,20]. This review synthesizes current knowledge on MNPs cerebral uptake routes, neurotoxic manifestations, and molecular pathways while evaluating therapeutic prospects [4,21]—including antioxidant, anti-inflammatory, neuroprotective, and nanomaterial-based strategies. We conclude by identifying key research challenges and future directions to advance understanding of MNPs-related neuropathology and mitigation approaches.

## 2. Sources and Exposure Pathways of Micro- and Nanoplastics

Micro- and Nanoplastics (MNPs) are plastic particles smaller than 5 mm, whereas NPs measure under 1 μm in diameter [22]. Most MNPs derive from the environmental breakdown of larger plastic debris. Their sources range from industrial and packaging materials to consumer products and wastewater treatment sludge [7]. These particles disperse across aquatic systems, soils, sediments, and the atmosphere, eventually reaching humans via drinking water and food chain accumulation [21].

**Figure 1:**
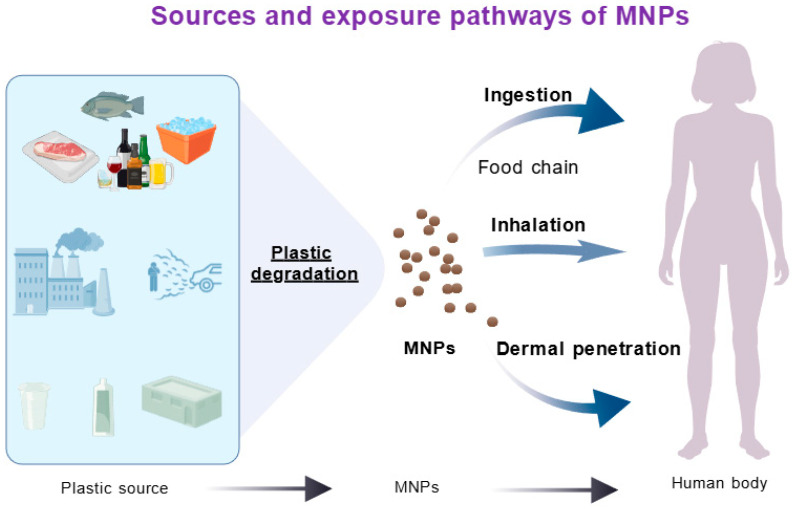
Sources and exposure pathways of micro/nano plastics.

Micro- and Nanoplastics (MNPs) enter the human body primarily through dietary intake, particularly via contaminated seafood [23], though inhalation and dermal exposure also contribute. The ubiquity of plastics has introduced additional exposure routes, including airborne particles from tire abrasion, textile fibers, and construction dust, which can penetrate deep into the lungs and enter systemic circulation [24,25]. MNPs have been identified in human feces, blood, placental tissue, meconium, and multiple organ samples [26]. These particles traverse biological barriers—including the intestinal epithelium, alveolar membrane, and blood–brain barrier—accumulating in distal organs such as the liver, spleen, lymph nodes, kidneys, and brain, where they may cause systemic toxicity [27]. The smaller size fraction of nanoplastics exhibits enhanced biodistribution potential, amplifying their health implications. This pervasive environmental presence and demonstrated bioaccumulation underscore MNPs’ capacity to induce neurotoxicity and other systemic health effects (Figure 1).

The assessment of human exposure to MNPs faces significant methodological challenges, with the reliability of detection results constrained by multiple factors. Technical difficulties persist in detecting MNPs within human samples, characterised by analytical heterogeneity. Current human exposure assessments suffer from inconsistent methodologies and inadequate quality control, resulting in poor data comparability and hindering accurate quantification of actual exposure levels. The absence of globally harmonised standards for extraction, identification, and quantification severely limits data comparability across studies. Furthermore, plastic contamination readily contaminates samples and experimental procedures, potentially compromising result accuracy and reliability. Comprehensive characterisation of MNPs in human samples—including particle size, morphology, polymer type, and surface chemistry—presents substantial difficulties. Consequently, MNPs detection in human samples suffers from considerable uncertainty and a lack of standardised analytical methodologies.

Establishing standardised methods for determining MNPs levels in biological samples is a fundamental prerequisite for effectively monitoring human MNP exposure and assessing associated health risks. Currently, quantitative and analytical protocols in this field remain under development, with differentiated technical approaches often employed for MNPs of varying compositions. For instance, inductively coupled plasma mass spectrometry (ICP-MS) serves as the gold standard for metal or metal oxide MNPs [28]; polymeric MNPs frequently establishing standardised methods for determining MNP levels in biological samples is a fundamental prerequisite for effectively monitoring human MNP exposure and assessing associated health risks. Currently, quantitative and analytical protocols in this field remain under development, with differentiated technical approaches often employed for MNPs of varying compositions. For instance, ICP-MS serves as the gold standard for metal or metal oxide MNPs; polymeric MNPs frequently rely on pyrolysis-gas chromatography/mass spectrometry (Py-GC/MS), Raman imaging, and fluorescence-labelled quantification techniques [29]. Researchers including Koelmans and Qiu have established quality evaluation standards for MNPs analysis in freshwater and drinking water, encompassing sampling, sample preparation, laboratory conditions, and quality control. Spectroscopic and spectro-analytical methods provide foundational support for exposure assessment. However, an integrated analytical technique capable of simultaneously obtaining particle size, morphology, and mass concentration information, while offering high specificity and low detection limits, remains elusive. Consequently, developing systematic and reliable standardised detection protocols for MNPs in biological samples remains a critical challenge requiring urgent breakthroughs in this field.

## 3. Micro- and Nanoplastics-Mediated Neurotoxicity

The CNS exhibits heightened vulnerability to MNPs. Although the BBB restricts most foreign substances, MNPs can translocate to distal brain regions, where their accumulation promotes oxidative stress, neuroinflammation, suppressed acetylcholinesterase activity, mitochondrial dysfunction, and disrupted autophagy. These cascading effects ultimately compromise neuronal integrity and cognitive function [30,31]. Experimental evidence from diverse models confirms NP-induced neurotoxicity: in Caenorhabditis elegans, nanoparticles trigger lipofuscin deposition and apoptotic cell death [32,33], while maternal exposure in mice facilitates transgenerational NPs transfer with concomitant neurological impairment. In non-mammalian zebrafish models, NPs have also been demonstrated to permeate and accumulate within developing embryos, altering neuronal and glial marker genes such as neuronal G1 (Neurog1) and glial fibrillary acidic protein (Glial Fibrillary Acidic Protein, Gfap) [34], thereby inducing neurotoxicity. Evidently, MNPs can cross species barriers to mediate neurotoxicity.

Micro- and Nanoplastics (MNPs), as a complex class of pollutants, often exhibit significant variations in their physical and chemical properties. These differences manifest across multiple aspects, including particle size, morphology, surface area, surface charge, crystallinity, and chemical composition [35,36]. Several studies have compared the neurotoxicity of MNPs differing in size, shape, surface charge, and chemical composition, suggesting that various physicochemical properties influence MNP neurotoxicity [37,38]. These include polymer type, surface modification, particle size and morphology, and degree of ageing. Research generally indicates that amino-modified particles interact more readily with cells, while smaller, fibrillar particles more easily penetrate the blood–brain barrier. Positively charged MNPs may exhibit stronger interactions with cell membranes, leading to heightened toxicity [6,39] (Table 1). Aged particles enhance oxidative stress and adsorption capacity, exerting a more pronounced effect on neurotoxicity.

### 3.1. Potential Pathways for Micro- and Nanoplastics to Enter the Brain and Induce Neurotoxicity

The nervous system represents a primary target for MNP toxicity, with multiple routes facilitating their entry into the brain and subsequent neurotoxic effects. These pathways encompass trans-BBB translocation, olfactory uptake via nasal inhalation, gut–brain axis transport, and systemic distribution through lymphatic and circulatory networks.

#### 3.1.1. Penetration of the Blood–Brain Barrier

While the BBB effectively blocks most large molecules and particles from reaching the brain, the small size and toxic nature of MNPs allow them to circumvent this protective barrier [46]. Moreover, MNPs can significantly increase the permeability of the BBB. In vivo studies have demonstrated that following seven consecutive days of nanoparticle injection, BBB permeability markedly increased, with dose-dependent accumulation observed within the mouse brain [47]. Measurements of blood–brain barrier permeability following MNP exposure can typically be conducted using multiple methodologies. These include in vivo tracer techniques, such as determining dye concentration in brain tissue after intravenous injection of tracers like Evans blue or fluorescent dextran [48,49]; in vivo imaging methods, such as contrast-enhanced magnetic resonance imaging; and in vitro BBB models to measure trans-endothelial resistance values and tracer permeability rates [50]. Currently, human-derived “blood-brain barrier chips” are also employed for quantitative assessment of vascular permeability [51]. Additionally, biomarker detection methods may be utilised, such as monitoring changes in the intracerebral levels of plasma proteins normally unable to cross the BBB (e.g., albumin, IgG), to indirectly evaluate alterations in blood–brain barrier permeability. These particles not only traverse the BBB but also bind to neuronal protein fibers, triggering neurotoxicity that may elevate Parkinson’s disease risk [52]. MPs can breach the BBB within two hours, inducing neuroinflammation, disrupting neural function, and potentially accelerating neurodegenerative disorders including Alzheimer’s and Parkinson’s diseases [53].

#### 3.1.2. Nasal Inhalation

Micro- and Nanoplastics (MNPs) can bypass the BBB through inhalation, initially depositing in the lungs before reaching the brain via retrograde transport along the nasal cavity [17]. Experimental evidence confirms this pathway, with 80 nm NPs detected in murine brains following intranasal administration or aerosol exposure [7]. Researchers from Freie Universität Berlin and the University of São Paulo identified the olfactory nerve as a vulnerable point in the BBB, creating a direct conduit for MNPs [54].

#### 3.1.3. Gut–Brain Axis

The gut–brain axis connects these organs through multiple pathways, including the autonomic nervous system, Hypothalamic–pituitary–adrenal axis (HPA axis), enteric nervous system, gut barrier, gut-associated lymphoid tissue, and microbiome [55]. Gut health profoundly influences brain function [56,57], with studies linking MNP exposure to neurotoxicity via this axis. For instance, deficiency of the key antioxidant transcription factor Nrf2 in the gut exacerbates MNP-induced neurotoxicity, demonstrating a direct gut-nervous system connection. Plastic particles disrupt the microbiota–gut–brain axis [58], while gut inflammation can propagate CNS inflammation [59]. MNPs may adsorb bacterial Lipopolysaccharide (LPS), enhancing microglial uptake and neuroinflammation [60]. Notably, as the core neural pathway of the gut–brain axis, MNPs can even accumulate within the vagus nerve, directly inducing neurodegeneration [61]. Furthermore, circadian rhythm-related pathways mediated by the gut–brain axis have also been implicated in the neurotoxic processes of NPs [39]. Based on these findings, modulating gut–brain axis function may represent an intervention strategy to inhibit MNP-induced neurotoxicity. For instance, common gut modulators such as melatonin and probiotic supplements could be employed [62]. Melatonin stabilises the intestinal environment and protects the barrier through receptor-mediated and antioxidant pathways, acting in a top-down manner [63]. Probiotics, conversely, exert a ‘bottom-up’ effect by altering microbial community composition, training the immune system, and generating beneficial metabolites, thereby enhancing gut function [64,65]. Both approaches may demonstrate potential application value in preventing MNP-induced neurotoxicity.

#### 3.1.4. Lymphatic and Circulatory Systems

The intestinal lymphatic system can transport MNPs into circulation, potentially impacting the CNS [66]. Although the precise lymphatic mechanisms remain unclear, this pathway represents another route for brain exposure. Circulating MNPs may be phagocytosed by macrophages that subsequently infiltrate the brain, where high-resolution imaging reveals MPs lodged in cortical vasculature, disrupting local blood flow [67]. Endothelial cells may also internalize bloodborne MNPs, compromising BBB integrity and permitting direct parenchymal entry, where they interact with neurons and glia to trigger neurotoxicity [68].

### 3.2. Potential Mechanisms of Micro- and Nanoplastics-Induced Neurotoxicity

Research continues to elucidate the pathways by which MNPs enter organisms and their underlying neurotoxic mechanisms. Current evidence points to four primary mechanisms: direct physical damage, chemical toxicity, immune-mediated inflammation, and cellular dysfunction [69,70]. Key biological pathways include mitochondrial impairment, oxidative stress, autophagosomal disruption, and non-BBB penetration routes [71]. These mechanisms interact synergistically, collectively contributing to neural cell stress and the development of neurodegenerative and cognitive disorders.

#### 3.2.1. Cytotoxic Mechanisms of Micro- and Nanoplastics

- Micro- and Nanoplastics-Induced Neurotoxicity Through Cytotoxicity

The cytotoxic effects of MNPs exhibit dose-dependent relationships with intracellular accumulation, mediated through oxidative stress, membrane disruption, immune activation, and DNA damage [72,73]. Nanoparticles readily undergo endocytosis due to their size, accumulating intracellularly to trigger direct or indirect stress responses—a pivotal factor in their cytotoxicity [74]. Experimental data demonstrate that NPs promote pro-inflammatory cytokine release and excessive Reactive oxygen species (ROS) generation, establishing oxidative stress conditions [75]. Mitochondrial dysfunction frequently follows, impairing energy metabolism while amplifying ROS production in a self-perpetuating cycle [7]. Supporting this model, pharmacological inhibition of endocytosis reduces polystyrene nanoparticle (PS-NP) uptake and consequent toxicity. Microplastics exert cytotoxic effects primarily through enhanced ROS generation and inflammatory cascades [76].

Multiple cell models corroborate MNP-induced cytotoxicity. Primary neuronal cultures exhibit apoptosis following MNP exposure, while PS-NPs activate the Autophagy pathway—Adenosine Monophosphate-Activated Protein Kinase/Autophagy-Linking Kinase 1 (AMPK/ULK1) pathway, inducing mitophagy in SH-SY5Y cells (Human neuroblastoma cell line cells) and dopaminergic neurons [42,43]. HepG2 cells (Hepatocellular carcinoma cell line) treated with 50 nm PS-NPs show oxidative stress, diminished antioxidant defenses, and 25–48% cell death. Busch et al. observed membrane damage, lysosomal rupture, and metabolic suppression in Caco-2 (Human colorectal adenocarcinoma cell line) cells exposed to similar nanoparticles [77,78,79]. Cellular defense mechanisms, including lysosomal clearance and exocytosis, partially counteract MNP accumulation. However, neuronal susceptibility to stress renders even acute exposures potentially neurotoxic, with irreversible consequences. Collectively, MNP cytotoxicity operates through interconnected pathways, with cellular-level damage ultimately manifesting as systemic neurotoxicity.

ii. Micro- and Nanoplastics-Induced Inflammatory Response Mediates Neurotoxicity

Micro- and Nanoplastics (MNPs) activate the brain’s resident immune cells, particularly microglia, initiating a cascade of neuroinflammatory processes. These particles directly stimulate microglia to release pro-inflammatory cytokines such as tumor necrosis factor-α (TNF-α) and interleukin-6 (IL-6), exacerbating local inflammation and neurotoxicity. Activated glial cells have been shown to internalize MNPs—for example, PS-NPs accumulate in murine microglia, triggering inflammatory disturbances in astrocytes and oligodendrocytes that collectively impair neuronal activity [80]. PS-NP exposure induces reactive astrocytosis and elevates lipid-binding protein-2 (Lipocalin-2)secretion, a lipid carrier protein that promotes neuronal death, suggesting neurotoxicity may arise from neuronal stress or astrocyte-derived neurotoxins [81]. NPs also penetrate cells, activating inflammasomes and stimulating inflammatory factor release [82], while simultaneously disrupting antioxidant defenses, increasing intracellular Fe^2+^, and inducing lipid peroxidation to amplify inflammation. In mice, MP exposure exacerbates hippocampal inflammation and disrupts dendritic spine density [26]. Both PS-MP-treated human microglia and murine brain tissue exhibit Nuclear factor κB signalling pathway (NF-κB pathway) activation, elevated pro-inflammatory cytokines, apoptotic markers, and altered microglial differentiation markers, corroborating inflammatory activation [47,83].

Micro- and Nanoplastics (MNPs) further modulate neuroinflammation through gene regulatory mechanisms. Transcriptomic analysis of PS-MP-exposed (Human Glial Cell Line) HMC-3 cells demonstrates altered expression of immune-related gene clusters, immunoglobulins, and microRNAs, indicating genetic-level regulation [84].

The gut–brain axis critically mediates MNP-induced neuroinflammation [85]. Single-cell RNA sequencing reveals increased Interleukin-1 beta (IL-1β)-positive gut macrophages following NP exposure. Chronically exposed mice develop cognitive and memory deficits linked to neuronal/oligodendrocyte degeneration, microglial activation, and cerebral T helper 17 cell (Th17 cell) accumulation [57,86]. These impairments may partly originate from gut macrophage-derived IL-1β, representing a key pathway for NP-mediated neuroinflammation [87].

Collectively, In experiments involving MNPs and neural tissue, the immune response within neural tissue may be assessed through observation of glial cell activation, detection of inflammatory cytokine levels, analysis of inflammation-related signalling pathways, and examination of the gut–brain axis. MNPs drive neuroinflammation through glial activation, inflammasome stimulation, genetic modulation, and gut–brain interactions, ultimately causing neuronal damage and functional decline.

#### 3.2.2. Specific Biological Pathways of Micro- and Nanoplastics-Induced Neurotoxicity

- Micro- and Nanoplastics mediate neurotoxicity by inducing oxidative stress and mitochondrial dysfunction

Exposure to magnetic nanoparticles can induce excessive production of reactive oxygen species within mitochondria, triggering oxidative stress and impairing mitochondrial function. This leads to a decline in mitochondrial membrane potential and mitochondria-mediated apoptosis. Dysfunctional mitochondria, in turn, further increase ROS generation, thereby forming a self-amplifying vicious cycle that ultimately heightens susceptibility to neuronal diseases. Exposure to MNPs elevates neuronal susceptibility to disease by triggering oxidative stress. ROS serve as central mediators of neurotoxicity, manifested through ferroptosis, free radical generation, and disruption of the antioxidant system. Excessive ROS production, resulting from the imbalance between intracellular oxidants and antioxidants, promotes lipid peroxidation and subsequent neuronal cytotoxicity [88]. Oxidative stress biomarkers include altered activities of antioxidant enzymes such as glutathione peroxidase 4 (GPx4) and superoxide dismutase (SOD), alongside elevated levels of lipid peroxidation products like malondialdehyde (MDA) [89]. Studies consistently link MNP exposure to heightened ROS levels; for example, Schirinzi et al. observed significantly increased ROS in brain and epithelial cell models following polyethylene (PE) and polystyrene (PS) particle exposure compared to controls [90]. In Wistar rats, ROS production rose dose-dependently after MNP exposure [91]. Cultured neuron experiments further revealed that PS-NP accumulation induced oxidative stress, while HDAC6 inhibitor-mediated NP clearance reduced cytoplasmic accumulation and toxicity [92]. Current methods for assessing MNP-induced oxidative damage to neural tissue include: Direct detection of ROS using fluorescent probes such as DCFH-DA; measuring the activity of antioxidant enzymes such as superoxide dismutase, catalase, and glutathione peroxidase; detecting oxidative stress biomarkers including lipid peroxidation products, protein glycation products, and 8-hydroxy-2′-deoxyguanosine; and analysing the expression of genes and proteins associated with the Nrf2/ARE pathway [93,94]. These methods collectively constitute a multi-tiered research framework for evaluating MNP-induced neuro-oxidative damage.

Beyond direct oxidative damage, MNP-induced ROS initiates complex signaling cascades that exacerbate toxicity. These pathways include p53, MAPK, and Nrf2 signaling [95,96]. Their coordinated activation or suppression establishes an intricate network through which MNPs exert oxidative stress-mediated neurotoxicity.

Mitochondria represent a critical target for MNPs. These particles disrupt mitochondrial function through direct or indirect mechanisms, initiating downstream signaling cascades where oxidative stress features prominently. MNPs accumulate within mitochondria, impairing electron transport chain integrity, damaging mitochondrial membranes, and destabilizing membrane potential, which results in depolarization. Such disturbances not only reduce ATP synthesis efficiency but also stimulate excessive free radical generation [91,97], precipitating cellular damage through DNA lesions, protein oxidation, lipid peroxidation, and antioxidant system depletion [98,99]. This represents a hypothetical theoretical mechanism for MNPs-mediated mitochondrial dysfunction inducing neurotoxicity, which requires further validation. Although larger MPs may not penetrate mitochondrial membranes directly, they induce oxidative stress and Na+/K+ channel dysregulation, indirectly compromising mitochondrial function.

Mitochondrial dysfunction exhibits a well-established association with ferroptosis, a necrotic cell death process driven by mitochondrial alterations. MNP exposure aggravates oxidative stress by suppressing antioxidant pathways, thereby promoting ferroptosis and mitophagy. In rats, maternal nanopolystyrene exposure induces hippocampal ferroptosis and mitophagy via the p53 pathway [91], impairing offspring neurodevelopment. BV2 microglial cells exposed to NPs demonstrate inflammation and ferroptosis mediated by the c-Jun N-terminal Kinase/Heme Oxygenase-1/Ferritin Heavy Chain 1 (JNK/HO-1/FTH1) axis, alongside pro-inflammatory cytokine release, underscoring the role of mitochondrial dysfunction in microglial neurotoxicity [100].

ii. Micro- and Nanoplastics-Mediated Neurotoxicity via Neurotransmitter Disruption

Neurotransmitters regulate neuronal communication, with their homeostasis being essential for proper brain function. MNPs entering the brain inhibit acetylcholinesterase (AChE) activity, inducing behavioral alterations, as demonstrated by reduced AChE levels in mice following seven-day exposure to PS-NP aerosols [101]. Similar AChE suppression occurs in Alzheimer’s and Parkinson’s diseases. NPs also alter vesicular catecholamine storage, diminish exocytotic spike frequency, reduce neurotransmitter release per event, and impair vesicle membrane fusion [102]. MNPs interference with neurotransmitter systems operates through multiple mechanisms, including membrane structural disruption, enhanced endocytosis, oxidative stress induction, inflammatory activation, and direct modulation of synthesis-related enzymes. Experimental evidence suggests MNPs may specifically target tryptophan hydroxylase, catechol-O-methyltransferase, and tyrosine hydroxylase—key enzymes governing serotonin and dopamine production [103,104]. Although investigations continue, the hypothesis linking MNPs-induced neurotoxicity to neurotransmitter dysregulation has gained substantial empirical support.

iii. Micro- and Nanoplastics-Mediated Neurotoxicity via Disruption of Cell Signaling Pathways

Exposure to MNPs disrupts multiple intracellular signaling pathways, a central mechanism underlying their neurotoxic effects. These disturbances form an intricate regulatory network that governs neuronal responses and toxicological outcomes.

Micro- and Nanoplastics (MNPs) activate oxidative stress pathways through NADPH oxidase and mitochondrial electron transport chain stimulation, generating excessive ROS [105,106]. The resulting ROS initiates downstream cascades including p53-mediated cell cycle and apoptosis regulation, MAPK family pathways governing stress responses and apoptosis, Nrf2-driven antioxidant defenses, and Phosphatidylinositol 3-kinase/Protein Kinase B (PI3K/Akt)-dependent survival signaling [106,107]. Inflammatory pathways show marked activation, particularly NF-κB, which promotes immune cell stimulation and pro-inflammatory cytokine release (e.g., TNF-α, ILs), exacerbating neuroinflammation and neuronal damage [83]. The MAPK pathway contributes to oxidative stress, inflammation, and apoptosis simultaneously. Mitochondrial-mediated apoptosis, involving B-cell lymphoma-2 gene/protein (Bcl-2) family proteins and caspase activation, represents another critical pathway for MNP-induced neuronal injury. MNPs also impair blood–brain barrier integrity by disrupting its maintenance pathways, facilitating neurotoxicant penetration.

**Figure 2:**
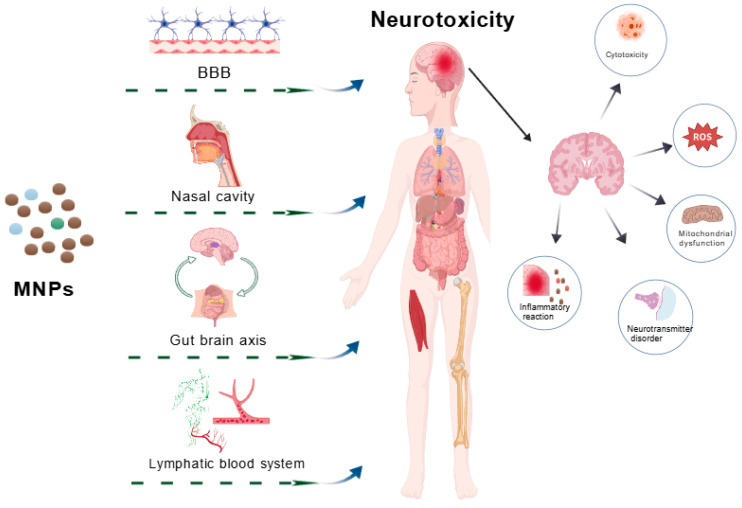
The pathways and possible mechanisms of neurotoxicity induced by micro/nano plastics. Microplastics/nanoplastics induce brain inflammation, cytotoxicity, mitochondrial dysfunction, neurotransmitter disorders, oxidative stress, and other neurotoxicity through the blood–brain barrier, nasal inhalation, gut–brain axis, lymphatic and hematological systems. Abbreviation: MNPs, micro and nanoplastics; BBB, blood–brain barrier; ROS, reactive oxygen species.

Micro- and Nanoplastics (MNPs) modulate autophagy homeostasis via Mechanistic Target of Rapamycin (mTOR) and PI3K/Akt pathways [108,109] and disrupt neurotransmitter systems, including cholinergic and glutamatergic signaling, impairing neuronal communication [108]. This widespread interference with signaling networks creates complex, unpredictable toxicological outcomes. Elucidating pathway cross-talk remains essential for understanding MNPs neurotoxicity. MNPs additionally alter energy metabolism, endocrine function, and synaptic activity [80,83], contributing to cognitive deficits and other neurological impairments (Figure 2).

## 4. Micro- and Nanoplastics in Neurodegenerative Diseases: Research Progress and Therapeutic Options

Micro- and Nanoplastics (MNPs) may contribute to the onset and progression of neurodegenerative diseases such as Parkinson’s disease (PD) and Alzheimer’s disease (AD) by triggering protein aggregation, neuroinflammation, and oxidative stress in the nervous system. Emerging studies aim to elucidate the link between MNPs-induced neurotoxicity and neurodegeneration while investigating potential preventive and therapeutic strategies.

### 4.1. Micro- and Nanoplastics and Parkinson’s Disease

Parkinson Disease (PD), a chronic neurodegenerative disorder, involves dopaminergic neuron loss and pathological α-synuclein accumulation in brain neurons [110]. Both in vitro and in vivo studies suggest MNPs may exacerbate PD pathogenesis. PS-NPs, for instance, promote α-synuclein nucleation, elevating PD risk [111]. PS-NPs also impair gut–brain axis development, worsening PD pathology [111]. Neurotransmitter dysregulation represents a key mechanism of MNPs neurotoxicity; MNPs inhibit AChE activity upon brain entry, inducing behavioral alterations. Notably, reduced AChE activity occurs in PD, implying MNPs may accelerate disease progression through analogous pathways. Regarding treatment options, these predominantly involve pollution control strategies, research into toxicity-alleviating substances, and so forth. Additionally, several novel therapies are currently under investigation, such as immunotherapy targeting α-synuclein, small-molecule drug interventions, gene therapy and cell replacement, and mitochondrial function protection. However, these approaches can only delay the progression of PD by addressing downstream pathological mechanisms, whilst the direct elimination of MNPs remains a significant challenge.

### 4.2. Micro- and Nanoplastics and Alzheimer’s Disease

Alzheimer’s Disease (AD), characterized by progressive memory decline and cognitive impairment, features amyloid plaque deposition and neurofibrillary tangle formation [112]. Although its etiology remains incompletely understood, environmental factors play a pivotal role. Research indicates that biological and environmental MNPs substantially increase AD incidence. Polystyrene nanoparticles, even at low concentrations, accelerate amyloid-β (Aβ) nucleation and oligomer formation, inducing pronounced neurotoxicity [113].

The potential risks of MNPs in AD have garnered significant attention. Current research is exploring methods to mitigate their potential hazards and treat AD from multiple angles. Notably, studies have demonstrated that nanotechnology can enable precise clearance of pathological proteins, overcome the blood–brain barrier for drug delivery, and serve as contrast agents for magnetic resonance imaging (MRI) and drug delivery systems [114]. For instance, Notably, research has demonstrated that nanotechnology can precisely eliminate pathological proteins and overcome the blood–brain barrier for drug delivery, serving as both a contrast agent for MRI and a drug delivery system [115]. This reflects that the biological effects of nanoparticles depend on their physicochemical properties, application context, and dosage. Environmental or unintentionally exposed NPs are typically unmodified and biocompatible. They may compromise blood–brain barrier integrity through mechanisms such as oxidative stress, inflammatory responses, and mitochondrial dysfunction, thereby inducing abnormal Aβ aggregation and excessive tau phosphorylation, which promotes neurodegenerative disease. Therapeutic nanoparticles, however, are precisely engineered and frequently modified to possess “targeting” capabilities, thereby minimising toxicity and maximising therapeutic efficacy.

### 4.3. Micro- and Nanoplastics and Other Neurodegenerative Diseases

Following cellular internalisation, MNPs can disrupt neuronal homeostasis through mechanisms including the induction of oxidative stress, mitochondrial dysfunction, and chronic neuroinflammation. These processes are recognised as pivotal stages in the onset and progression of neurodegenerative diseases. The preceding discussion has primarily examined the association between MNPs and PD and AD, with current research on MNP-induced neurodegeneration predominantly focused on these two conditions. Furthermore, studies suggest links between MNPs and other neurological disorders, such as amyotrophic lateral sclerosis (ALS) and spinal muscular atrophy (SMA). However, evidence remains limited in areas including Huntington’s disease and multiple sclerosis. MNPs may also participate in disease progression through epigenetic regulation. Research indicates that MNPs can induce epigenetic alterations including abnormal DNA methylation, altered histone modifications, and dysregulation of non-coding RNAs. These changes may lead to reduced synaptic stability and sustained transcriptional reprogramming, thereby increasing susceptibility to diseases such as ALS [116]. For instance, polystyrene nanoparticles induce intracellular oxidative stress, promoting abnormal aggregation of the TAR DNA-binding protein 43 kDa (TDP-43) and subsequently triggering ALS-like pathological phenotypes. This demonstrates, at the molecular level, the potential mechanism by which MNPs contribute to neurodegenerative processes [117], both studies demonstrate the pivotal role of MNPs in the pathogenesis and progression of neurodegenerative diseases.

In summary, the potential hazards of MNPs to human neurological health have become apparent, revealing a certain correlation with neurodegenerative diseases. However, the causal relationship remains to be confirmed. While current animal and cellular experiments support preliminary findings, it is common practice in toxicological studies to test at high doses or non-physiological concentrations. For instance, concentrations in in vitro experiments are typically 100 times higher than the average concentration found in human blood [118,119]. Nevertheless, exposure to excessive MNPs poses challenges for assessing human risk relevance. Rigorous human studies to validate these findings are currently lacking. Consequently, no evidence to date establishes a causal link between MNP exposure and huma AD or PD, reflecting limitations in existing models.

### 4.4. Preventive and Therapeutic Measures for Micro- and Nanoplastics-Induced Neurotoxicity

Research into the prevention and control of MNP pollution is currently primarily conducted through experimental exploration in biological models. For instance, prior studies have demonstrated that trehalose mitigates neurotoxicity induced by polystyrene nanoplastics exposure by enhancing microbe–gut–brain axis function [120]; further research indicates that activating the ErbB4 receptor alleviates neuropathology and cognitive impairment caused by polystyrene microplastics, thereby offering a potential therapeutic strategy against MNP-related neurotoxicity [121]. Within experimental settings, the core challenge in evaluating the efficacy of such therapeutic interventions lies in systematically comparing multi-level differences among the “treatment group (MNPs exposure combined with intervention)”, the “exposure-only group (MNPs exposure alone)”, and the “control group”. Specifically, at the molecular and cellular levels, interventions must be quantitatively assessed for their reversal of MNP toxicity pathways through indicators such as reduced blood–brain barrier permeability, decreased oxidative stress and inflammatory markers, and diminished glial cell activation [83,122,123]. At the neurofunctional level, standardised behavioural tests like the Morris water maze and open field assay should objectively confirm improvements in learning, memory, and emotion-related behaviours [37,124]. At the tissue and structural level, techniques such as quantitative neuronal survival analysis and immunofluorescence imaging of synaptic markers should be employed to visually validate cellular and ultrastructural morphological repair. This multidimensional assessment system, integrating biochemical indicators, behavioural performance, and morphological evidence, provides a systematic and robust scientific basis for evaluating the efficacy of experimental prevention and treatment strategies.

Global initiatives have implemented source control, process management, and technological innovation to address MNP pollution. Current strategies emphasize reducing plastic consumption, improving waste treatment methods, and promoting polymer recycling through assimilation techniques, though global waste management remains inadequate. Given the pervasive nature of plastic exposure, primary mitigation approaches include restricting single-use plastics, advancing biodegradable or durable alternatives, and employing high-temperature food processing. These measures may lower MNPs uptake and subsequent neurotoxic effects. Effective prevention and treatment require not only source regulation but also intervention in downstream pathological pathways.

## 5. Limitations of Current Evidence and Challenges in Translation

Currently, research into the neurotoxicity of MNPs continues to face a series of critical limitations, hindering the reliable translation of findings into human health risk assessments. Primary challenges include:Weak epidemiological evidence, with a lack of prospective population studies directly linking MNP exposure to neurological health outcomes; existing evidence is largely confined to cross-sectional surveys;Significant disconnect between model systems and real-world exposure scenarios, as most in vitro studies employ high-dose exposures far exceeding physiological concentrations and predominantly use unaged, surface-clean, monodisperse polystyrene particles, failing to reflect the complex ageing characteristics of environmental MNPs; Human biomonitoring data suffer from insufficient reliability. The absence of standardised pre-processing methods and analytical workflows, coupled with susceptibility to background contamination during experiments, results in poor comparability between studies and hinders accurate quantification of actual human exposure levels. Consequently, although existing model studies provide mechanistic insights into the potential neurotoxicity of MNPs, their ecological relevance and extrapolation value to humans remain subject to significant uncertainty. Overcoming these translational bottlenecks urgently requires methodological standardisation, the development of exposure models more closely approximating real-world environments, and the implementation of systematic population cohort studies.

## 6. Existing Research Issues and Future Outlook

Micro- and Nanoplastics (MNPs) contamination represents a pressing environmental challenge with unavoidable human exposure. While the BBB offers partial protection, studies demonstrate that MNPs can bypass this barrier via the gut–brain axis, nasal inhalation, and other routes, accumulating in neural tissues and posing neurotoxic risks. This underscores the urgency of improving source containment and risk mitigation. Current mechanistic insights remain limited, primarily addressing broad biological processes like cytotoxicity, inflammation, and oxidative stress without clarifying specific pathway targets. Further research must investigate MNPs neurotoxicity under realistic exposure scenarios, particularly their contribution to neurodegenerative disease progression. Methodological constraints also persist, as in vitro systems fail to replicate the in vivo microenvironment, and interspecies differences limit translational relevance. Furthermore, research indicates that MNPs pose extensive systemic health risks. Whilst this paper focuses on their toxic effects on the central nervous system, a comprehensive physiological perspective underscores the critical importance of conducting systematic risk assessments for MNPs in the future. Currently, comprehensively determining the systemic health risks of MNPs typically requires integrated in vivo toxicological studies [125]. These encompass the biodistribution and pharmacokinetic characteristics of MNPs within major organs; haematological and clinical biochemical indicators, such as analysis of hepatic and renal function and relevant inflammatory markers; histopathological examination of primary organs; neurofunctional assessment based on behavioural testing; and long-term observational studies addressing chronic toxicity and potential carcinogenicity [6,126].

Achieving clear observation of the MNPs and neural tissue is a crucial step towards elucidating their toxicological mechanisms. Presently, a suite of advanced imaging techniques offers new avenues for research in this field: ultra-high-resolution optical microscopy techniques, such as stimulated emission depletion microscopy and structured illumination microscopy, enable nanometre-scale precise localisation of fluorescently labelled MNPs; advanced electron microscopy techniques like cryo-electron microscopy can resolve in situ interaction details between MNPs and biological membranes; Raman and infrared spectral imaging can capture chemical fingerprint information of MNPs, revealing their composition and distribution; furthermore, synchrotron-based X-ray fluorescence microscopy and phase-contrast imaging techniques enable high-sensitivity elemental distribution and structural imaging in a label-free manner. This underscores their potential to replace or complement conventional methods (such as the confocal microscopy employed in this study) in future research, thereby advancing investigations into MNPs neurotoxicity mechanisms towards higher resolution and more comprehensive dimensions. clear observation of the microscopic interactions between MNPs and neural tissue is a crucial step towards elucidating their toxicological mechanisms. Presently, a suite of advanced imaging techniques offers new avenues for research in this field: ultra-high-resolution optical microscopy techniques, such as stimulated emission depletion microscopy and structured illumination microscopy, enable nanometre-scale precise localisation of fluorescently labelled MNPs; advanced electron microscopy techniques like cryo-electron microscopy can resolve in situ interaction details between MNPs and biological membranes; Raman and infrared spectral imaging can capture chemical fingerprint information of MNPs, revealing their composition and distribution; furthermore, synchrotron-based X-ray fluorescence microscopy and phase-contrast imaging techniques enable high-sensitivity elemental distribution and structural imaging in a label-free manner. This underscores their potential to replace or complement conventional methods (such as the confocal microscopy employed in this study) in future research, thereby advancing investigations into MNPs neurotoxicity mechanisms towards higher resolution and more comprehensive dimensions. Future work should prioritize sensitive MNPs detection technologies, robust statistical frameworks, and validated biological models to strengthen evidence-based interventions.

## 7. Conclusions

Micro- and Nanoplastics (MNPs) enter the human body through ingestion, inhalation, and dermal absorption, subsequently reaching the brain via the BBB, olfactory pathways, and gut–brain axis. Cerebral accumulation elicits multifaceted molecular responses, including BBB disruption, oxidative stress, neuroinflammation, acetylcholinesterase inhibition, mitochondrial impairment, and autophagic dysfunction. Emerging evidence suggests MNPs may modulate neurotoxicity-related gene expression, contributing to protein misfolding, neuronal depletion, neurotransmitter dysregulation, and behavioral abnormalities that exacerbate neurodegenerative and neurodevelopmental disorders.

Advancing neuroprotective strategies requires elucidating precise MNP toxicity mechanisms and developing targeted interventions. Current efforts concentrate on pollution source reduction, yet effective detoxification methods remain elusive. Investigating downstream pathological targets will inform evidence-based risk prevention and therapeutic development. Concurrent priorities include refining high-sensitivity detection assays, characterizing bioaccumulation dynamics, evaluating chronic low-dose effects, examining pollutant interactions, and optimizing experimental models to comprehensively assess MNPs health impacts.

## Figures and Tables

**Table 1:** Research on MNPs-Mediated Neurotoxicity.

Model Source	Biological Model	Solution	Mechanism of Action and Practical Significance	Refs.
in vivo model	*Mammalian mouse*	Three different sizes of fluorescent PS-MPs were used to investigate the accumulation of PS-MPs in the brains of mice following oral administration.	Microglial phagocytosis of polystyrene microplastics induces immune alterations and apoptosis in vivo	[20]
	*Caenorhabditis elegans*	MNP exposure	ROS overproduction, lipofuscin accumulation and dopaminergic neuron loss	[32]
	*Zebrafish*	Directly inject MNPs into aquaculture water or add MNPs to feed, then expose zebrafish to the suspension.	Increased levels of oxidative stress and apoptosis in zebrafish brains, with behavioural experiments detecting impaired memory and learning abilities alongside reduced acetylcholinesterase activity.	[40]
	*Zebrafish*	Directly inject MNPs into aquaculture water or add MNPs to feed, then expose zebrafish to the suspension.	NPs have also been demonstrated to penetrate and accumulate within developing embryos, altering the expression of neuronal and glial marker genes such as Neurog1 and Gfap, thereby inducing neurotoxicity.	[34]
	*Human samples*	Fluorescently labelled polystyrene particles were administered orally (e.g., via oral gavage, drinking water exposure, or intragastric inoculation). The particle size range was between 0.04 μm and 20 μm.	Fluorescently labelled polystyrene particles are absorbed by the body; upon oral ingestion, these particles are detected in both the intestines and the brain.	[41]
in vitro model	*Primary cultured neuronal cells*	Magnetic Nanoparticle Exposure	The AMP-activated protein kinase/autophagy-light chain kinase 1 (AMPK/ULK1) pathway induces excessive mitochondrial autophagy in differentiated SH-SY5Y cells and dopaminergic neurons.	[42,43]
	HepG2 Cell	Exposure to 50nm PS-NPs	Induces oxidative stress, diminishes antioxidant capacity, and results in cell death rates as high as 25–48%.	[44]
	Caco-2 Cell	Exposure to 50nm PS-NPs	Cell membrane disruption, lysosomal lysis, and a marked reduction in cellular metabolic activity	[45]

## Data Availability

No new data were created or analyzed in this study. Data sharing is not applicable to this article.
